# Supplementary material for: Splice-Junction-Based Mapping of Alternative Isoforms in the Human Proteome
Source: Cell Rep. Author manuscript; Available in PMC 2020 Jan 15. (PMC6961840; doi:10.1016/j.celrep.2019.11.026)
Supplement: 2 [file NIHMS1546469-supplement-2.zip › DF1/PXD010154/Lung/Lung_39_MAPKAPK5_QVIEEQTTSHESTGGAK.pdf]

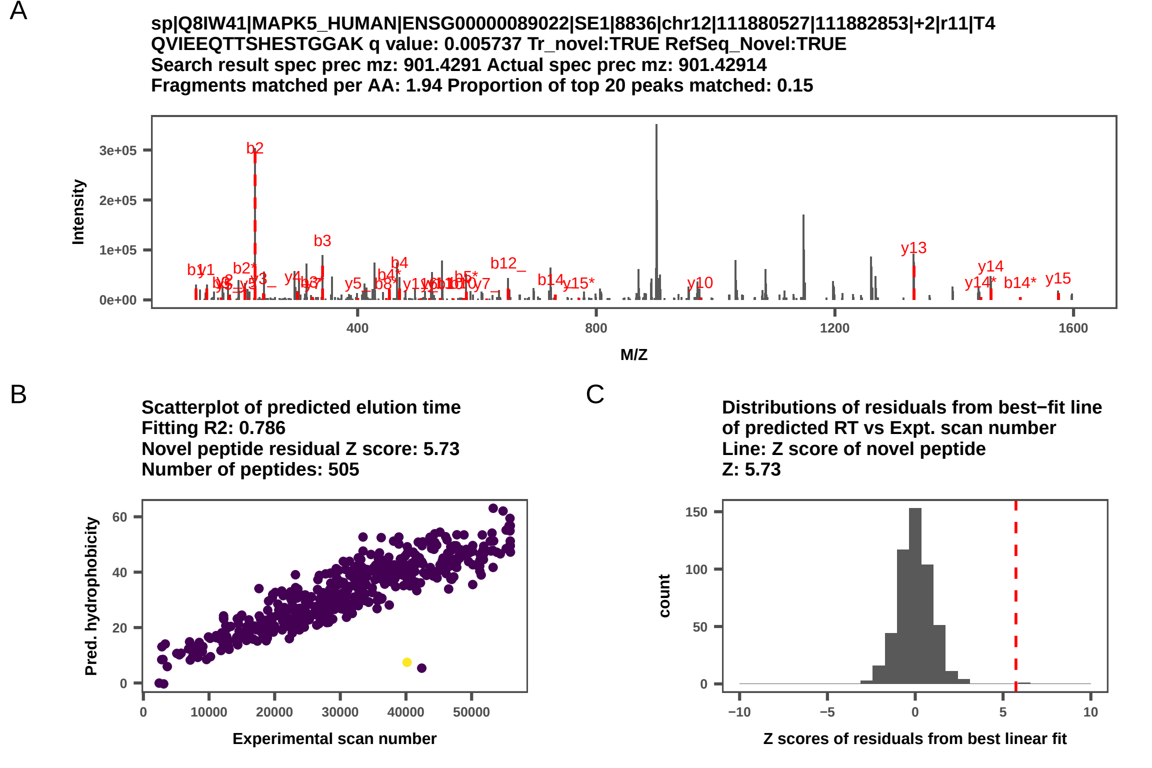
A
sp|Q8IW41|MAPK5_HUMAN|ENSG00000089022|SE1|8836|chr12|111880527|111882853|+2|r11|T4
QVIEEQTTSHESTGGAK q value: 0.005737 Tr_novel:TRUE RefSeq_Novel:TRUE
Search result spec prec mz: 901.4291 Actual spec prec mz: 901.42914
Fragments matched per AA: 1.94 Proportion of top 20 peaks matched: 0.15
Intensity
3e+05
2e+05
1e+05
0e+00
b2
b3
b4
b12_
y13
y14
y14*
b14*
y15
y10
b14
y15*
b5*
y4
y5
b4*
b8*
y7
400
800
1200
1600
M/Z
B
Scatterplot of predicted elution time
Fitting R2: 0.786
Novel peptide residual Z score: 5.73
Number of peptides: 505
Pred. hydrophobicity
60
40
20
0
0
10000
20000
30000
40000
50000
Experimental scan number
C
Distributions of residuals from best−fit line
of predicted RT vs Expt. scan number
Line: Z score of novel peptide
Z: 5.73
count
150
100
50
0
−10
−5
0
5
10
Z scores of residuals from best linear fit
